# High-precision mapping reveals rare *N*^*6*^-deoxyadenosine methylation in the mammalian genome

**DOI:** 10.1038/s41421-022-00484-1

**Published:** 2022-12-27

**Authors:** Li-Qian Chen, Zhang Zhang, Hong-Xuan Chen, Jian-Fei Xi, Xue-Hong Liu, Dong-Zhao Ma, Yu-Hao Zhong, Wen Hui Ng, Tao Chen, Daniel W. Mak, Qi Chen, Yao-Qing Chen, Guan-Zheng Luo

**Affiliations:** 1 MOE Key Laboratory of Gene Function and Regulation, Guangdong Province Key Laboratory of Pharmaceutical Functional Genes, State Key Laboratory of Biocontrol, School of Life Sciences, Sun Yat-sen University, Guangzhou, Guangdong China; 2 Guangdong Cardiovascular Institute, Medical Research Center, Guangdong Provincial People’s Hospital, Guangdong Academy of Medical Sciences, Guangzhou, Guangdong China; 3 School of Biomedical Sciences, LKS Faculty of Medicine, The University of Hong Kong, Hong Kong, China; 4 School of Public Health (Shenzhen), Sun Yat-sen University, Shenzhen, Guangdong China

**Keywords:** DNA methylation, Methylation analysis

## Abstract

*N*^6^-deoxyadenosine methylation (6mA) is the most widespread type of DNA modification in prokaryotes and is also abundantly distributed in some unicellular eukaryotes. However, 6mA levels are remarkably low in mammals. The lack of a precise and comprehensive mapping method has hindered more advanced investigations of 6mA. Here, we report a new method MM-seq (modification-induced mismatch sequencing) for genome-wide 6mA mapping based on a novel detection principle. We found that modified DNA bases are prone to form a local open region that allows capture by antibody, for example, via a DNA breathing or base-flipping mechanism. Specified endonuclease or exonuclease can recognize the antibody-stabilized mismatch-like structure and mark the exact modified sites for sequencing readout. Using this method, we examined the genomic positions of 6mA in bacteria (*E. coli*), green algae (*C. reinhardtii*), and mammalian cells (HEK239T, Huh7, and HeLa cells). In contrast to bacteria and green algae, human cells possess a very limited number of 6mA sites which are sporadically distributed across the genome of different cell types. After knocking out the RNA m^6^A methyltransferase METTL3 in mouse ES cells, 6mA becomes mostly diminished. Our results imply that rare 6mA in the mammalian genome is introduced by RNA m^6^A machinery via a non-targeted mechanism.

## Introduction

DNA modifications have been discovered and extensively studied for decades^1^. Among dozens of known forms of DNA modifications, 5-methylcytosine (5mC) is well-characterized for its diverse functions in transposon suppression, genomic imprinting, X-chromosome inactivation, and other crucial cellular processes^2,3^. *N*^6^-deoxyadenosine methylation (also referred to as 6mA or m^6^dA) is another form of DNA modification pervasively found in prokaryotes and also present in a small group of unicellular eukaryotes^4–7^. Recent studies reported the presence of 6mA in *Drosophila* and *C. elegans* and described its dynamics and potential functions. This has generated great research interest in 6mA as a new epigenetic marker in more complex eukaryotes^8–12^. Subsequent studies have detected the presence of 6mA in a variety of eukaryotes, including plants, vertebrates, and even mammals^13–17^. Together, these discoveries have extended the boundaries in our traditional understanding of 6mA, therefore having the potential to further expand the existing pool of known epigenetic markers found in eukaryotes.

Despite this, researches on the identification of 6mA and functional studies are few and there is currently limited evidence to support 6mA as an epigenetic regulator in higher eukaryotes^18^. The global 6mA levels determined by liquid chromatography-mass spectrometry (LC-MS/MS) varied greatly in mammals, ranging from relatively high (comparable to 5mC) to undetectable levels^19–21^. Even though LC-MS/MS is regarded as a gold standard to detect and quantify nucleotide modifications, the sequence information of the modification remains unknown; thus, contamination from the environment or human intervention may generate artificial signals^20,21^. In addition, most recent studies have proposed that trace *N*^6^-methyladenine is misincorporated into DNA via the nucleotide-salvage pathway, which recycles the modified adenosine from RNA degradation metabolites^22,23^. On the other hand, whereas certain studies argue that 6mA is highly dynamic and significantly elevated during specific biological processes such as embryogenesis or DNA damage response, others argue that the global 6mA levels are extremely low in most circumstances^15,24–27^.

Compared to LC-MS/MS, sequencing-based methods identify modified bases affiliated with their sequence context, which facilitates a comprehensive genome-wide analysis along with downstream functional studies^28^. Methylated DNA immunoprecipitation (IP) coupled with deep sequencing (MeDIP-seq) has become the most popular method to profile DNA modifications (such as 6mA) and it works by taking advantage of the antibody’s specific affinity to enrich modified DNA fragments^29,30^. However, concerns have been raised that bias from the antibodies’ specificity may generate false-positive signals and overestimate the 6mA stoichiometry in some samples^31^. For species with a low 6mA level, the false-positive rate was unprecedentedly high to the extent that severely affected subsequent functional analysis. A recent report estimated a large proportion of peaks (50%–99%) could be false positives due to the non-specific binding of antibodies^31^.

Alternatively, some restriction enzymes can determine the modification status at a specific site with high sensitivity and specificity^32^. However, genome-wide approaches based on restriction enzymes are limited to only a subset of genomic regions with consensus sequence motifs^28^. Third-generation sequencing techniques, including, PacBio Single-Molecule Real-Time sequencing (SMRT-seq) and Oxford Nanopore Technologies (ONT), allow the detection of genome-wide DNA modifications at single-base resolution. Though third-generation sequencing has been applied successfully to bacteria and green algae, the high error rates and prerequisite of known consensus sequence hamper its application in detecting sites with low levels of modification present in plants and mammals^33^. Collectively, it is crucial to develop a sensitive and reliable mapping method for determining the presence of 6mA as well as the precise location of genuine 6mA sites in the genome, especially for organisms with very low 6mA levels.

In this study, we worked on developing an unbiased method to map genomic 6mA sites comprehensively and precisely in multiple species. Within double-stranded DNA, bases on one strand usually pair with complementary bases along the other strand. Surprisingly, we found that 6mA-specific antibodies could recognize the modified adenine embedded on the DNA duplex. After examining the chemical properties of 6mA, we speculate that the presence of 6mA attenuates the base-stacking and destabilizes the local DNA duplex^34,35^. We verified that the *N*^6^-deoxyadenosine base on duplex DNA promotes a mismatch-like structure, probably via a DNA breathing or base-flipping mechanism. This unique mismatch-like structure allows 6mA to be captured by 6mA-specific antibodies and subsequently recognized by certain nucleases or chemical cleavage reactions^36–39^. Following this principle, we developed a new mapping method MM-seq (modification-induced mismatch sequencing). We applied this method in bacteria and green algae and obtained precise genome-wide 6mA maps at single-base resolution. We then detected a limited number of 6mA sites and examined the distribution pattern in multiple human cell lines (HEK293T, Huh7, and HeLa cells). Notably, the non-overlap distribution of the rare 6mA sites in different technical and biological replicates suggested a non-targeted mechanism for 6mA biogenesis.

To further test this, we knocked out the RNA m^6^A methyltransferase METTL3 in mouse embryonic stem cells (mESCs) and compared the 6mA sites identified by MM-seq to that of wild-type mESCs. The 6mA sites in *Mettl3* knockout cells, which were originally limited, were further diminished. This supports the perspective that the rare presence of 6mA in the mammalian genome might be dependent on the RNA m^6^A pathway^22,23^. Not only do we expect MM-seq to serve as a high-precision method for mapping 6mA sites in organisms with a relatively high level of 6mA modification, but also to provide an insight into the controversy surrounding the presence and distribution of 6mA and other rare DNA modifications in more extensive biological systems.

## Results

### Antibody-binding to 6mA induces a mismatch-like structure

**Fig. 1:**
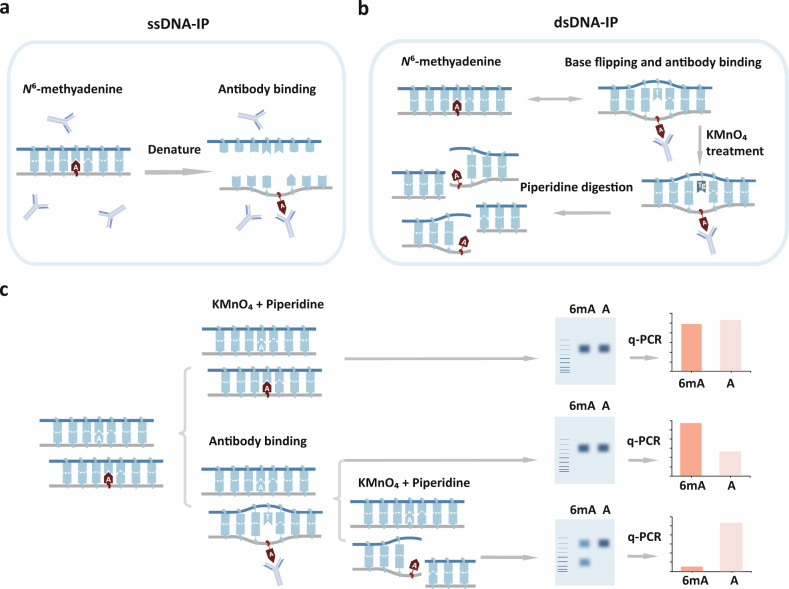
KMnO_4_ footprinting assay to detect the 6mA-induced mismatch-like structure. **a** The dsDNA is denatured prior to antibody binding during IP in the canonical MeDIP procedure. **b** Antibody captures transiently flipped *N*^6^-methyadenine base and releases the opposite thymine. The unpaired thymine can be oxidized by KMnO_4_ and subsequently digested by piperidine. **c** The products of KMnO_4_ footprinting assay can be quantified by q-PCR to indicate the presence of 6mA. Substrates with 6mA are prone to form a mismatch-like structure with the antibody binding and cleaved by piperidine.

The antibody specific to *N*^6^-methyladenine has been widely used to detect the presence of 6mA on DNA (Methylated DNA Immunoprecipitation, or MeDIP) and m6A on RNA (Methylated RNA Immunoprecipitation, or MeRIP). In MeDIP procedure for 6mA, the double-stranded DNA (dsDNA) has to be denatured in order to release the embedded A-T base pairing from the duplex for antibody binding (Fig. 1a). Unexpectedly, we found the 6mA immunoprecipitation using *Chlamydomonas reinhardtii* (*C. reinhardtii*) genomic dsDNA also yielded substantial products, and these enriched regions highly overlapped with peaks that were identified when using traditional DNA denaturing procedures (Supplementary Fig. S1a). We reasoned that the local region where 6mA locates might transiently melt, and the *N*^6^-methyladenine base would flip out of the dsDNA, therefore allowing it to be captured by the antibody. The captured *N*^6^-methyladenine base in its maintained flipped status releases the opposite thymine, thereby forming a mismatch-like structure (Fig. 1b).

To validate this hypothesis, we carried out the potassium permanganate (KMnO_4_) footprinting assay to detect mismatches^40^. First, we tested the system using two synthetic oligos that were complementary to each other except at one mismatched site. The unpaired thymine within the mismatch structure could be oxidized to thymine glycol by KMnO_4_ and then digested by piperidine^41^ (Supplementary Fig. S1b). We then conducted the KMnO_4_ footprinting assay using synthetic oligos with one 6mA site (Fig. 1b). After immunoprecipitation, KMnO_4_ treatment and piperidine digestion, the synthetic oligo with 6mA was cleaved in half while the oligo without 6mA remained intact (Supplementary Fig. S1c). We then used qPCR to quantify the products after piperidine treatment and found that the oligos with 6mA sites were much more sensitive to cleavage (~16 fold) than those without modification (Fig. 1c and Supplementary Fig. S1d). These results imply that the antibody facilitated the formation of a mismatch-like structure at the 6mA site, possibly via a DNA breathing or base-flipping mechanism (Fig. 1c).

### Detect 6mA sites by endonuclease cleavage

**Fig. 2:**
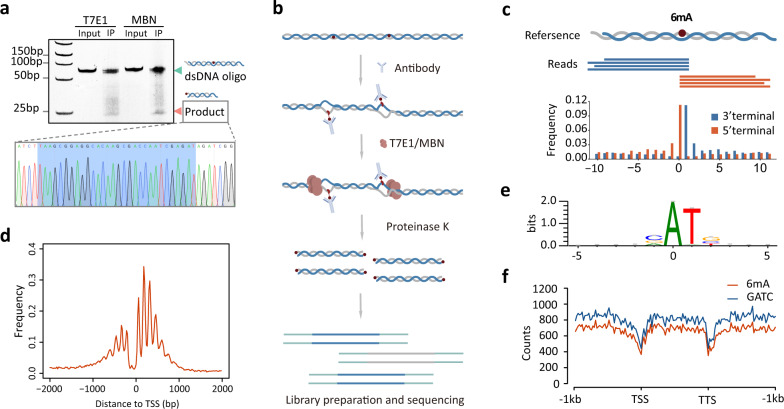
Development of endonuclease facilitated MM-seq method for 6mA mapping in *C. reinhardtii* and *E. coli*. **a** T7E1 and MBN endonuclease can recognize and digest 6mA sites in dsDNA oligos after immunoprecipitation. Sanger sequencing traces of the cleavage products are shown below. **b** A schematic diagram of MM-seq using T7E1 and MBN endonucleases. **c** Cleavage signals of reads mapped to the known 6mA sites in a genome-wide scale. **d** Accumulative plot of the distances between the 6mA sites and the transcription start sites (TSS). **e** Sequence logo showing VATB motif sequences of the 6mA sites detected in *C. reinhardtii*. **f** The distribution of the 6mA sites identified in *E. coli* displays a similar pattern to the genome-wide GATC motif. TSS transcription start site, TTS transcription termination site.

The explicit chemical cleavage signal at 6mA site inspired us to develop a genome-wide method for 6mA mapping. However, we noticed that the harsh reaction conditions of KMnO_4_ footprinting assay led to severe degradation of DNA molecules, thereby hindering the subsequent analysis such as DNA sequencing (Supplementary Fig. S1c). Therefore, we sought to identify a series of endonuclease which could recognize and cleave the mismatch-like structure while also capable of reacting in mild conditions. The T7 Endonucleases 1 (T7E1) is a structure-selective enzyme that recognizes and catalyzes the cleavage of DNA mismatches^42^; Mung Bean Nuclease (MBN) is another endonuclease that explicitly degrades single-stranded DNA^43^. We first verified their digestion efficiency using dsDNA oligos with mismatches (Supplementary Fig. S2a, b). We then synthesized 59-nt dsDNA with an internal 6mA site at the 25^th^ position and tested whether the T7E1/MBN could recognize the mismatch-like structure induced by antibody binding (Fig. 2a). After immunoprecipitation and endonuclease digestion, we found that both the T7E1 and MBN could digest the dsDNA to ~25-nt fragments. Sanger sequencing confirmed that the oligo was cleaved exactly at the 6mA site (Fig. 2a).

Given the endonuclease sensitivity in recognizing the mismatch-like structure at 6mA sites, we developed a new method termed MM-seq (modification-induced mismatch sequencing) for genome-wide 6mA mapping (Fig. 2b). The genomic DNA was first fragmented and then incubated with 6mA antibody, followed by IP and T7E1/MBN digestion. After proteinase K treatment, IP products were end-repaired and ligated to NGS adapters for high-throughput sequencing (Fig. 2b). To assess the effectiveness of MM-seq, we used *C. reinhardtii* as the model organism in which the 6mA atlas had been well-characterized. The sequencing reads showed a clear preference for adenine at the 5' terminal, meeting our expectation of more frequent cleavage events at 6mA sites (Supplementary Fig. S3a). Compared to background reads, we found that the cleavage signals were significantly enriched at the 6mA sites that were identified in a previous study^4^ (Fig. 2c). Sites with a significantly higher cleavage score than the background were identified as potential 6mA. Collectively, we identified 130,457 and 104,791 sites in T7E1 and MBN digested samples, with ~76% recurrent sites in both samples (Supplementary Fig. S3b). We therefore used the more sensitive enzyme T7E1 for the following analysis. The distances between the 6mA sites and transcription start sites (TSS) showed a characteristic period pattern that had been well-defined in *C. reinhardtii* (Fig. 2d). Approximately, 97% of the 6mA sites were located at the consensus sequence with a VATB (V = G/A/C, B = G/T/C) motif and this is consistent with findings from previous reports^4,16^ (Fig. 2e and Supplementary Table S1). A similar period pattern was observed for 6mA sites within each possible form of VATB motif, whereas no pattern was observed for non-VATB motif (Supplementary Fig. S3c).

To assess the general applicability of the MM-seq method in a broader range of species, we then applied it to investigate 6mA in the *E. coli* genome. We identified 36,625 6mA sites in total, the majority of which (~96%) were located within the GATC motif (Supplementary Table S2). Meanwhile, more than 96% GATC motifs in the genome were determined to be modified, consistent with previous understanding of the Dam methylase in *E. coli*^44^. The genome-wide distribution of 6mA was generally consistent with the genomic locations of GATC motif, without any preference towards specific chromosomal regions or gene context (Fig. 2f). Together, these data indicate that MM-seq as a comprehensive and unbiased method for genome-wide 6mA mapping.

### Detect 6mA sites by exonuclease digestion

**Fig. 3:**
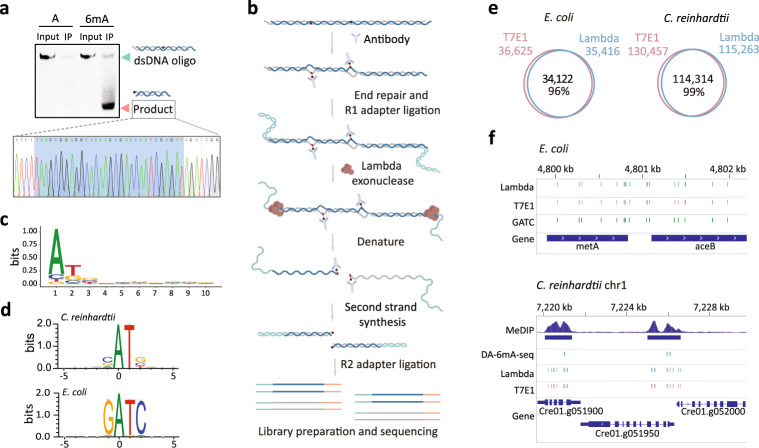
Exonuclease facilitated MM-seq and its application in multiple organisms. **a** A fragment resulting from Lambda exonuclease digestion of the synthetic oligo with a 6mA site. Sanger sequencing traces for the cleavage products is shown below. **b** A schematic diagram showing optimized MM-seq using the Lambda exonuclease. **c** Nucleotides composition at the 5’ terminal of NGS sequence reads. **d** Consensus sequences of the genomic context to the 6mA sites identified in *C. reinhardtii* and *E. coli*. **e** High number of overlapping 6mA sites detected between T7E1 endonuclease and Lambda exonuclease in *C. reinhardtii* and *E. coli*. **f** IGV illustration of 6mA sites identified in this study compared to published results from MeDIP-seq and DA-6mA-seq.

To further validate the reliability of MM-seq in an orthogonal manner, we sought to elucidate whether other nucleases could also recognize the 6mA-induced, mismatch-like structure. Lambda exonuclease catalyzes the degradation of nucleotides in dsDNA from the 5' end^45^. We speculated that the mismatch-like structure would hinder the Lambda exonuclease digestion along one strand of the dsDNA. Similar to the endonuclease digestion assay, we conducted the IP and exonuclease digestion reaction using Lambda exonuclease on the synthetic 59 bp dsDNA with an internal 6mA site (Fig. 3a). Here, we found that the length of the residual fragments indicated that the cleavage of the synthetic dsDNA occurred at the 6mA site. Furthermore, Sanger sequencing of the residual fragments revealed that the exonuclease digestion was blocked at the precise location where 6mA resided. In contrast, dsDNA oligo controls without the 6mA modification or those with the 6mA modification but without the IP step, did not produce any truncated product after exonuclease digestion (Fig. 3a). Together, our results show that both endonuclease and exonuclease enzymes can be used to identify mismatch-like structures associated with the base-flipping phenomenon resulting from 6mA modifications in dsDNA.

We further repeated the oligo digestion experiment using monoclonal antibodies from different vendors and found that the results were consistent, demonstrating the robustness of this reaction (Supplementary Fig. S4a–c). In addition, we showed that the Lambda exonuclease could accurately detect a single 6mA base on the single strand by using a semi-methylated dsDNA model, thus providing a feasible strategy to further distinguish the fully methylated (symmetric) from the semi-methylated (asymmetric) 6mA sites (Supplementary Fig. S4d).

We next applied exonuclease facilitated MM-seq to the mapping of the 6mA in *C. reinhardtii* and *E. coli* genomes (Fig. 3b). The first nucleotide at the 5' end of sequencing reads showed a distinct adenine bias, in line with the significantly attenuated exonuclease activity at the 6mA site (Fig. 3c). We set up a statistical model for testing the reliability of 6mA sites with a false-positive rate of less than 1%. Collectively, we identified ~110,000 6mA sites at the VATB motif and ~35,000 6mA sites at the GATC motif in *C. reinhardtii* and *E. coli*, respectively (Fig. 3d and Supplementary Table S3). Specifically, we observed a distinct periodic distribution pattern in *C. reinhardtii* that had been well-established in a previous report^4^ (Supplementary Fig. S5a). Over 89% of the 6mA sites were determined to be fully methylated in *E. coli*, among which ~94% resided in symmetric GATC motifs (Supplementary Table S2). These results agreed with the finding that the adenine nucleotide bases in GATC motifs were prevalently and exclusively methylated by the Dam enzyme in *E. coli*^44^. Notably, most of the 6mA sites (~96% and ~99% in *C. reinhardtii* and *E. coli*, respectively) were identified by using both the endonuclease and exonuclease facilitated MM-seq methods under comparable sequencing depth and statistical power (Fig. 3e, f and Supplementary Fig. S5b–e). Compared to the 6mA peaks identified using MeDIP-seq, over 95% of the 6mA sites determined by MM-seq were located within these peak regions (Fig. 3f).

To test the possible effects of other DNA modifications close to the 6mA site using MM-seq, we isolated genomic DNA from dam-/dcm- *E. coli* that was considered as absent of 6mA and 5mC. Then we artificially introduced 6mA to the GATC motifs using purified Dam methylase. The genomic DNA from wild-type *E. coli* contains 6mA on GATC motifs and 5mC on CCAGG motifs, while the artificially modified DNA from dam-/dcm- *E. coli* only contains 6mA on GATC motifs. We found that most of 6mA sites (> 81%) adjacent to 5mC sites were repeatable in both wild-type *E. coli* and that depleted of 5mC, indicating that other DNA modification had little influence to the 6mA detection by MM-seq (Supplementary Fig. S6a–c). Together, these results further confirm the high precision and robustness of MM-seq in mapping 6mA.

### 6mA sites are rare in mammalian cells and diminished after *Mettl3* knock-out

**Fig. 4:**
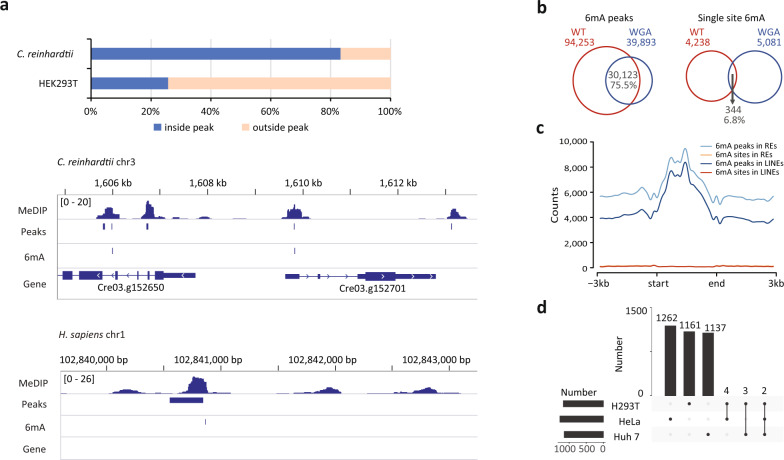
Application of MM-seq in mammalian cell lines. **a** The percentage of 6mA sites identified by MM-seq that fall within the 6mA peaks in *C. reinhardtii* and human HEK293T cells. The single-base 6mA sites and peaks are illustrated by IGV. **b** Venn diagrams showing 6mA peaks that were detected in HEK293T cells highly overlapped with those detected in WGA samples. **c** Accumulative distribution of the 6mA peaks and single-base 6mA sites at repetitive elements (REs) and LINEs. **d** Bar graph showing the total number of total 6mA sites that appear in each cell line and the total number of 6mA sites that are common between the cell lines as indicated by the dotted lines. Horizontal bars represent the total number of 6mA sites in each sample, and vertical bars with numbers represent the specific or common sites in one or more samples. The dotted lines indicate the site classification (specific or common).

Given the high precision of MM-seq, we then applied this method in mammalian cells (HEK293T) with low or undetectable 6mA levels. For the IP step, we supplemented a small amount of *C. reinhardtii* genomic DNA (gDNA) as spike-in positive controls against the human gDNA (1:20) to ensure data reliability. Here, we discovered that the majority (82%) of the 6mA sites of *C. reinhardtii* spike-ins corresponded with peak regions determined in MeDIP-seq. In comparison, even though numerous 6mA peaks were mapped to the human genome, only ~23% of these contained the single-base 6mA sites derived from MM-seq (Fig. 4a).

The high proportion of peaks without single-base information suggests a misleading peak calling procedure associated with conventional IP-based method, and this observation is in line with a previous report found that 50%–99% of the peaks were false positives due to the non-specific binding of antibodies^31^. We therefore used the MM-seq method on non-modified whole-genome amplified (WGA) DNA. Interestingly, we found that the 6mA peaks identified in the WGA samples largely overlapped with those identified in the WT samples (75.5%), which suggests the pervasiveness of false-positive peaks when using the conventional MeDIP-seq (Fig. 4b). In addition, we compared the detection of antibodies from SYSY (202003) and CST (56593 S), and found that 4148 and 3846 6mA sites in the human genome, among which they shared only 7 sites (Supplementary Fig. S4b, c). These results reinforced the notion that majority of the 6mA peaks that were previously reported using MeDIP-seq in humans were most likely to be false positives, which further suggests that data based only on antibodies should be treated with greater caution^20,21,31^.

Even though the single-base 6mA sites we identified in the human genome were rare, these limited sites may enrich at specific genomic regions and play an important regulatory role^15,26,46,47^. We therefore investigated whether 6mA sites have a tendency to cluster at specific regions, especially on repetitive elements (REs) of genome or mitochondrial DNA^15,48^. Indeed, we found that the IP-derived 6mA peaks were enriched at REs such as long interspersed nuclear elements (LINEs); however, after single-base site calling by MM-seq, this enrichment disappeared (Fig. 4c). We also noticed that a few 6mA sites were detected on the mitochondrial DNA, in line with previous reports^48^ (Supplementary Fig. S7a). METTL4 was previously reported to positively mediate mammalian mtDNA 6mA methylation. To further confirm the 6mA signal on mitochondrial DNA, we knocked down the putative 6mA methyltransferase METTL4 which was reported to be responsible for mitochondrial 6mA modification^48^. MM-seq results showed that the distribution of 6mA in knock-down cells was comparable to that in WT cells (Supplementary Table S4), suggesting that the limited sites on mitochondrial DNA were most likely to be false-positive. We speculated that the ultra-high sequencing depth and particular structure of mitochondrial DNA would introduce extra noise in 6mA identification. Moreover, we performed MM-seq simultaneously on three different cell lines (HEK239T, Huh7, and HeLa) to examine the recurrence of the single-base 6mA site in multiple cell types. Notably, individual 6mA sites appeared to be scattered throughout the genome of the different cell lines but sharing extremely few common sites (Fig. 4d).

To further investigate the presence of 6mA sites in mammals, we conducted MM-seq in mouse embryonic cells (mESCs). Similar to human cells, mESCs possessed a limited number (3806) of 6mA sites (Supplementary Fig. S7b). Moreover, even though the genome-wide distribution of 6mA was broadly scattered, the peaks indicated a depletion near the TSS region as previously reported^4^ (Supplementary Fig. S7c). We next performed MM-seq on the m^6^A deficient mESCs with the RNA m^6^A methyltransferase METTL3 knocked-out^49^ and showed that the number of 6mA sites was diminished to 779, approaching the detection limit of our method (Supplementary Fig. S7b). Collectively, these results support the perspective that the presence of rare 6mA is associated with the RNA m^6^A pathway in mammals^23^.

## Discussion

Preliminary studies revealed distinctive distribution patterns of 6mA in unicellular eukaryotes with characteristically high 6mA levels such as *C. reinhardtii* and *Tetrahymena*. The recent discoveries of the presence of 6mA in more complex eukaryotes, even in mammals, have placed 6mA under the spotlight. Nevertheless, the presence and biological significance of 6mA in eukaryotes have been the subject of intense debate as the lack of high-precision mapping methods has hindered functional and mechanistic studies of 6mA. A recent study developed a quantitative method that deconvolutes 6mA in samples of interest from contamination sources and suggested that a reassessment of 6mA in eukaryotic DNA is warranted^50^. To provide an insight into the controversies surrounding 6mA^19–23^, we developed a novel 6mA mapping method called MM-seq with genome-wide coverage and high precision. This method builds on the putative 6mA-facilitated base-flipping and mismatch-like structure. Here, we used a 6mA-specific antibody to trigger a mismatch-like structure at modified sites and following this, an enzymatic cleavage reveals the exact single-base 6mA site on the genome. We applied MM-seq to examine the 6mA distribution pattern in bacteria and green algae, and this has provided a highly accurate and comprehensive 6mA landscape, demonstrating the superiority of MM-seq to previous methods.

A striking discovery was that 6mA might spontaneously flip out from the DNA duplex to form an extrahelical base during transient DNA melting or breathing events without the assistance by any other auxiliary enzyme. We demonstrated that a specific antibody could stabilize these extrahelical bases to form a mismatch-like structure. On the other hand, these mismatch-like structures would transiently exist in the absence of antibody binding due to spontaneous flipping of the extrahelical base and would thus not be detected by nuclease recognition. It is worth noting that this method does not only detect 6mA but also the potentiality of other naturally occurring DNA damages, particularly when the level of 6mA is low.

Furthermore, our findings raised two interesting questions: (i) whether 6mA base-flipping also occurs in vivo and (ii) if any 6mA reader proteins function via the recognition of the transient base-flipping. Notably, a recent study showed that site-specific demethylation of the putative 6mA demethylase ALKBH1 explicitly occurs at unwound regions of the DNA bubble instead of single-stranded DNA (ssDNA) and dsDNA, thus raising the possibility that extrahelical 6mA is recognized and stabilized via an unknown mechanism in vivo^51^. More interestingly, in vitro experiments showed that the YTH domain of RNA m^6^A reader protein also binds to the 6mA site within the unwound regions of the DNA bubble^52^. Nevertheless, our results indicated that the 6mA level was extremely low in mammalian cells. Whether this base-flipping mechanism play an important role in a wider range of organisms or is restricted to unicellular organisms which are known to have characteristically high 6mA abundance remains to be determined.

Since the initial discovery of the presence of 6mA in mammals, researchers have devoted more towards understanding the regulatory role of this elusive DNA modification through the use of various methods. Our MM-seq method provides an orthogonal approach for comprehensive 6mA mapping with high precision. Very recent studies proposed that the genomic 6mA in mammals primarily originates from the nucleotide-salvage pathway, which recycles the modified nucleotides such as m^6^A in RNA^23^. Our results may support this finding that trace amounts of 6mA could be identified in the mammalian genome which rarely overlaps between different cell lines. The knock-out of the m^6^A writer gene *mettl3* further reduced the number of 6mA sites, presumably due to the reduced levels of modified nucleotide bases in the cell.

Other than the 6mA modification, there are various forms of DNA modification in the eukaryotic genome, some of which may play an important role in the regulation of the epigenome. Here, innovative mapping methods are required to better understand their functions. Notably, MM-seq can also be potentially used in the detection of DNA modifications other than 6mA, so long as specific antibodies that target these modified bases or binding proteins that facilitate the base-flipping of the modified base are employed, thereby giving rise to the mismatch-like structures. With continual efforts in optimizing the parameters of this technique, MM-seq holds great promise in the extensive mapping of various DNA modifications, particularly in the detection of extremely rare base modifications in organismal studies.

## Materials and methods

### Oligonucleotide synthesis

DNA oligonucleotides used in this study were synthesized from Sangon Biotech (Supplementary Table S5). Double-stranded DNA oligos were generated by annealing complementary forward and reverse strand oligos with a final concentration of 100 mM in annealing buffer (50 mM NaCl, 10 mM Tris pH 7.4). The mixture was incubated at 95 °C for 5 min, 85 °C for 2 min, 75 °C for 2 min, 65 °C for 3 min, 55 °C for 3 min, 45 °C for 2 min, 35 °C for 2 min and gradually cooling to below 30 °C.

### Materials preparation and genomic DNA isolation

*Chlamydomonas reinhardtii* strain CC-503 (cw92 mt+) were obtained from Chlamydomonas Resource Center (http://www.chlamycollection.org). *C. reinhardtii* cells were cultured in Tris Acetate Phosphate (TAP) medium with modified trace element solution (pH 7.5)^53^ under the continuous cool fluorescent white light of 40 to 60 µE m^−2^ s^−1^ at 110 rpm at 22 °C. Then the cells were treated with lysis buffer (50 mM Tris-HCl pH 8.0, 200 mM NaCl, 20 mM EDTA, 2% SDS, 1 mg/mL Proteinase K) and CTAB buffer (50 mM Tris-HCl pH 8.0, 20 mM EDTA, 1.4 M NaCl, 2% CTAB, 1% PVP4000) at 65 °C for 30 min. Total RNA was digested using 100 µg/mL RNase A for 20 min at room temperature. The genomic DNA of *C. reinhardtii* was isolated by phenol-chloroform extraction and ethanol precipitation. *E. coli* genomic DNA was extracted using FastPure Bacteria DNA Isolation Mini Kit (Vazyme, DC103).

Human cell lines were cultured in 10% (v/v) FBS (Gibco) containing DMEM medium (Corning) at 37 °C under 5% CO_2_ and showed negative in mycoplasma contamination testing. Mouse 129 embryonic stem cells (mESCs) and *Mettl3* knockout (KO) mESCs were provided by Dr. Jiekai Chen^49^ at the Guangzhou Institutes of Biomedicine and Health, Chinese Academy of Sciences. Cells were collected and then lysed by lysis buffer (0.1% TritonX-100, 0.1% Tween-20 diluted in 1×PBS) supplemented with 5 μg Proteinase K (Sangon) and 2 μL RNase A (Takara) under the condition of shaking (~1000 rpm) at 37 °C for 30 min. Then the MinElute PCR Purification Kit (QIAGEN, 28006) was used to isolate genomic DNA.

### KMnO_4_ footprinting

In total, 4 μL 160 mM KMnO_4_ solution was added to 10 μM 3' FAM labeled DNA oligos in a total volume of 40 μL, and then incubated at 30 °C until the color turned to brown. The reaction was stopped by adding 4.8 μL β-mercaptoethanol and mixed until the solution turned clear and transparent. After the mixture was purified using DNA Oligo Clean & Concentrator kit (Zymo Research), 10% (v/v) piperidine was added and incubated at 90 °C for 30 min. The sample was purified with DNA Oligo Clean & Concentrator kit, and visualized using 20% denaturing polyacrylamide gel.

A synthetic oligonucleotide chain with 6mA modification was used as the detection object, and human GAPDH gene was treated as the internal control. Equal amounts of dsDNA of A and 6mA were taken, respectively, and the same amount of internal reference was added to carry out IP at 4 °C overnight. The IP product was rinsed with 1×IP Buffer. Then the IP product was treated with 5 μL 40 mM KMnO_4_, and H_2_O was added to 50 μL. After purification, the product was cut with 10% piperidine. 1%–2% of the purified products were taken for qPCR quantification. The reaction system of 10 μL was prepared with the Cham SYBR qPCR Master Mix (Vazyme, Q311-02) solution and tested on Kubo Tech q225 machine.

### NGS library construction

During MM-seq with endonuclease digestion, 6mA immunoprecipitation reaction was conducted in the total volume of 500 μL with 4 μg fragmented gDNA or 200 μM 6 mA dsDNA oligo with 3' FAM fluorescent modification, 5 μL (0.5 mg/mL) anti-m^6^A/6mA antibody (56593S, CST and 202003, SYSY), 100 μL 5×IP Buffer, 30 μL pre-block protein-A magnetic beads. The mixture was transferred to a head-over-tail rotating wheel to incubate at 4 °C overnight and placed on a magnetic rack until beads were captured. The supernatant was discarded and the beads were washed with 500 μL ice-cold 1×IP Buffer by gentle vortex. The beads were re-suspended in the T7E1 or MBN digestion system (M0302S or M0250S, NEB) and incubated for 30 min at 37 °C. After washing with 500 μL ice-cold 1×IP Buffer, 5 μL Proteinase K was added to the beads and incubated for 2 h at 65 °C with 1000 rpm. The products were purified by Zymo Research oligo Clean & Concentrator and eluted in 12 μL ddH_2_O. We used 12% polyacrylamide gel to separate and verify products from oligos. NGS libraries were constructed for products from genomic DNA using NEBNext Ultra II DNA Library Prep Kit (E7645S).

The same procedure for 6mA immunoprecipitation reaction was applied when using exonuclease cleavage in MM-seq. The beads with IP products were subjected to primer extension, end-repair, adapter ligation and primer extension steps using T4 DNA polymerase (M0203S, NEB), T4PNK (T4 Polynucleotide Kinase, M0201S, NEB), T4 DNA ligase (M0202S, NEB) and Phi29 DNA polymerase (M0264S, NEB), respectively. The beads were washed by 500 μL ice-cold 1×IP Buffer after each treatment. The beads were re-suspended in the Lambda exonuclease (M0262S, NEB) digestion system and incubated for 30 min at 37 °C. After washing with 1×IP Buffer, the beads were treated by 5 μL Proteinase K to digest antibody. The mixture was incubated for 2 h at 65 °C with 1000 rpm speed. Then the beads were spun down briefly and placed the tube on magnetic rack for 30 s until beads were captured and transferred the supernatant into a new tube. The products were purified by Zymo Research oligo Clean & Concentrator and elute in 12 μL ddH_2_O. After primer extension by Phi29 DNA polymerase, the products were added into R2 adapter ligation reaction. Then, the products were cleaned up using 52 μL well-mixed AMPure beads and the dsDNA fragments were eluted in 30 μL ddH_2_O. After the PCR amplification, the library was purified using 50 μL well-mixed AMPure beads or agarose gel with size selection.

### NGS data analyses

Sequencing adapters were firstly removed from raw reads using cutadapt (v. 1.15). Clean reads were mapped to the reference genome (Cre_v5.5 for *Chlamydomonas*, ensemble bacteria release-45 for *E. coli* and hg19 for human) using bowtie2 (v 2.3.3) with default parameters except one mismatch. The sorted bam file was generated using samtools (v1.6). Picard (v1.129, https://broadinstitute.github.io/picard/) and MarkDuplicates were used to mark and remove duplicates. Integrative Genomics Viewer (IGV) was used for data visualization. Reads were also mapped to the mycoplasma genome to confirm not being contaminated (Supplementary Table S6).

For MM-seq data using T7E1/MBN endonuclease, we discarded single-end mapping results and counted both 5' and 3' terminals of each fragment as potential T7E1 digest sites. For MM-seq data using Lambda exonuclease, only 5' terminal of sequenced fragments was calculated. A binominal distribution model was assumed that each fragment could be randomly sheared or cleaved by structure-sensitive enzymes. The *P*-values were further corrected by Bonferroni correction, and FDR ≤ 0.01 was set under the coverage cutoff 5. The distance between each detected 6mA site and transcriptional start site (TSS) was calculated and plotted. The flanking sequences of 6mA sites were extracted, and WebLogo (v.3.6.0) was used to describe the base enrichment. When analyzing MM-seq for human and mouse samples with *C. reinhardtii* as spike-in, we firstly mapped and extracted clean reads belonging to *C. reinhardtii*. The remaining reads were then mapped to human or mouse genomes. 6mA peak calling for MeDIP-seq or MM-seq was conducted using MACS2 with *q*-value threshold of 0.01 (v2.1.1) and overlapping peaks were obtained using bedtools intersect (v2.26.0). R package chipseeker was used to annotate 6mA peaks or sites. Bed files of repetitive elements were downloaded from USCS. The blacklists for human (hg19) and mouse (mm10) were downloaded from https://github.com/Boyle-Lab/Blacklist. 6 mA sites and peaks located in regions of blacklists were removed using bedtools intersect.

### Accession codes

Data were deposited in NCBI’s Gene Expression Omnibus (GEO) under accession number GSE171698.

## Supplementary information

Supplementary Information
