# Supplementary material for: High-precision mapping reveals rare N6-deoxyadenosine methylation in the mammalian genome
Source: Cell Discov. 2022 Dec 27;8:138. doi: 10.1038/s41421-022-00484-1 (PMC9794812; doi:10.1038/s41421-022-00484-1)
Supplement: Supplementary file 1 — Supplementary Information [file 41421_2022_484_MOESM1_ESM.pdf]

Supplementary Information for

High-precision mapping reveals rare N^6-deoxyadenosine methylation in the mammalian genome

Li-Qian Chen[1,2,#], Zhang Zhang[1,#], Hong-Xuan Chen[1], Jian-Fei Xi[1], Xue-Hong Liu[1], Dong-Zhao Ma[1], Yu-Hao Zhong[1], Wen Hui Ng[1], Tao Chen[1], Daniel W. Mak[3], Qi Chen[4], Yao-Qing Chen[4], and Guan-Zheng Luo[1,*]

1 MOE Key Laboratory of Gene Function and Regulation, Guangdong Province Key Laboratory of Pharmaceutical Functional Genes, State Key Laboratory of Biocontrol, School of Life Sciences, Sun Yat-sen University, Guangzhou 510275, China

2 Guangdong Cardiovascular Institute, Guangdong Provincial People's Hospital, Guangdong Academy of Medical Sciences, Guangzhou 510060, China

3 School of Biomedical Sciences, LKS Faculty of Medicine, The University of Hong Kong, Hong Kong, China

4 School of Public Health (Shenzhen), Sun Yat-sen University, Shenzhen 518107, China

#These authors contributed equally

*Correspondence to luogzh5@mail.sysu.edu.cn

This file includes:

Figures. S1 to S7

Tables S1 to S6

Supplementary Figures

Figure S1. Antibody-binding to 6mA induces mismatch-like structure. a) 6mA peaks identified by MeDIP-seq using denatured DNA (ssDNA) and native DNA (dsDNA). Example of selected region is shown in IGV. b) Products of $KMnO_4$ footprinting assay. Synthetic dsDNA with the mismatches is subjected to cleavage. M indicates marker. M1 or M2 indicates oligo with one or two mismatches, respectively. c) $KMnO_4$ footprinting assay using synthetic dsDNA with A and 6mA sites. d) q-PCR melting curves of the products from $KMnO_4$ footprinting assay.

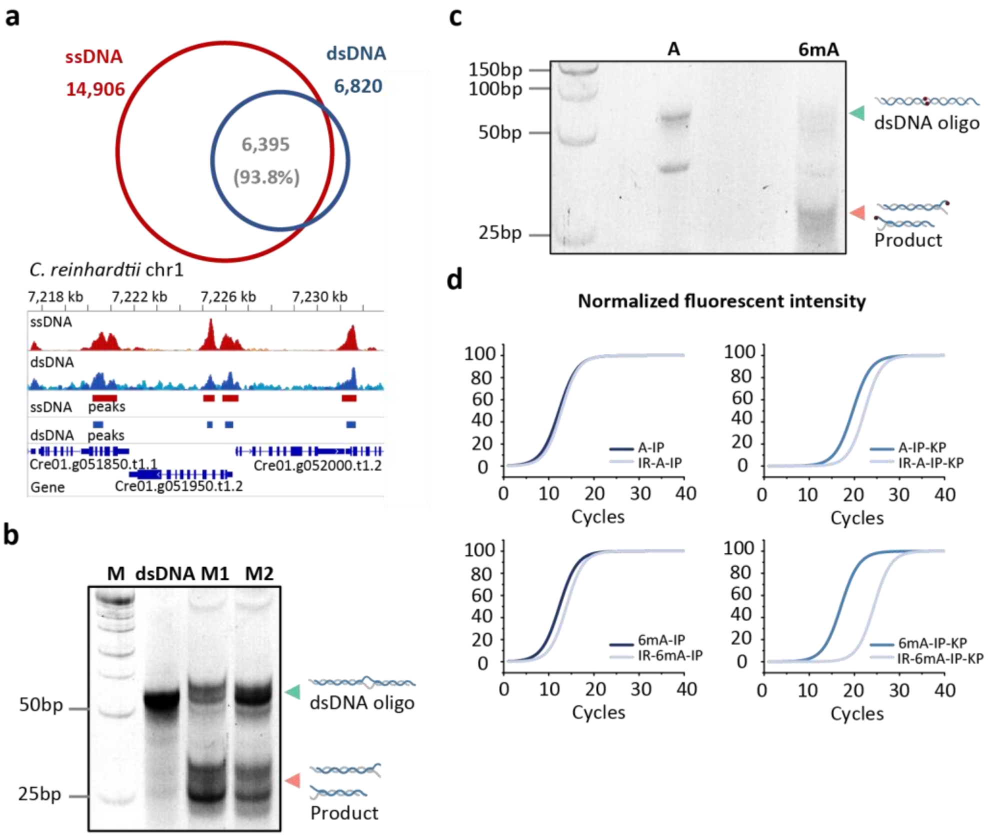

Figure S2. Verifications of endonuclease digestion on synthetic dsDNA oligos. The oligos are designed to contain 1 (M1), 2 (M2), or 4 (M4) mismatches. T7E1 (a) and MBN (b) endonucleases are used.

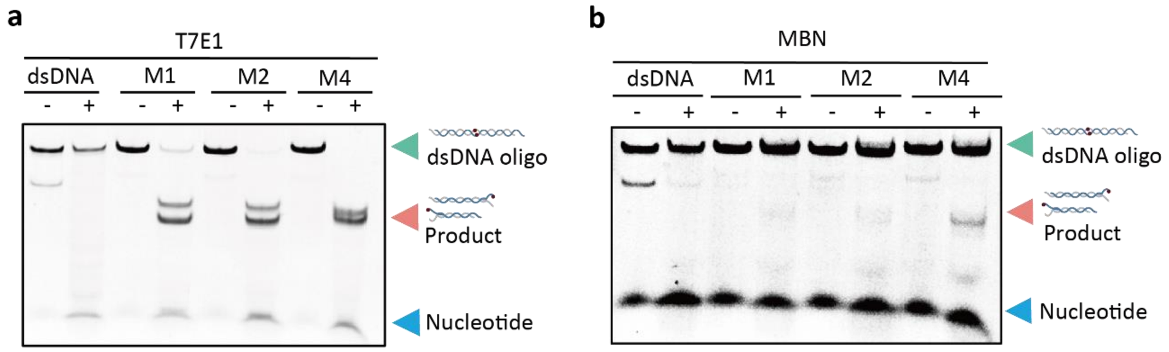

Figure S3. 6mA sites detected by MM-seq using T7E1 and MBN endonucleases. a) Nucleotide preference of the 5′ terminal. b) Common 6mA sites identified using two endonucleases. c) Accumulative distribution of 6mA sites around transcription start sites (TSS) in *C. reinhardtii*. 6mA sites within different motifs are plotted shown separately. d) Accumulative distribution of symmetric and asymmetric 6mA sites around transcription start sites (TSS) in *C. reinhardtii*. 6mA sites were detected using lambda exonuclease. 6mA sites within symmetric AT motif and asymmetric 6mA sites are separately plotted.

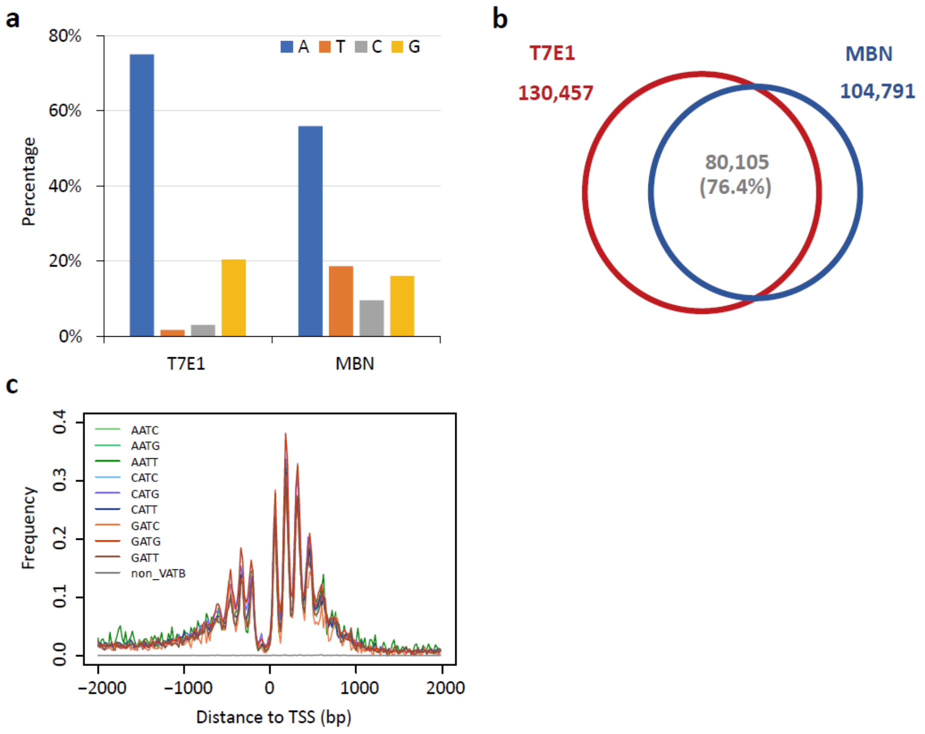

Figure S4. Products of Lambda exonuclease digestion on synthetic dsDNA oligos. a) Products of Lambda exonuclease digestion after immunoprecipitation. Anti-6mA antibodies from two different vendors (CST #56593S and SYSY 202003) are compared. b) The percentages of motifs for detected 6mA sites using different antibodies. c) Intersection of detected 6mA sites. d) Products of Lambda exonuclease digestion using oligos with semi-methylated 6mA site (Asymmetry) and fully-methylated 6mA site (Symmetry). Different protein beads (protein A beads and protein G beads) are used and compared in the immunoprecipitation procedure.

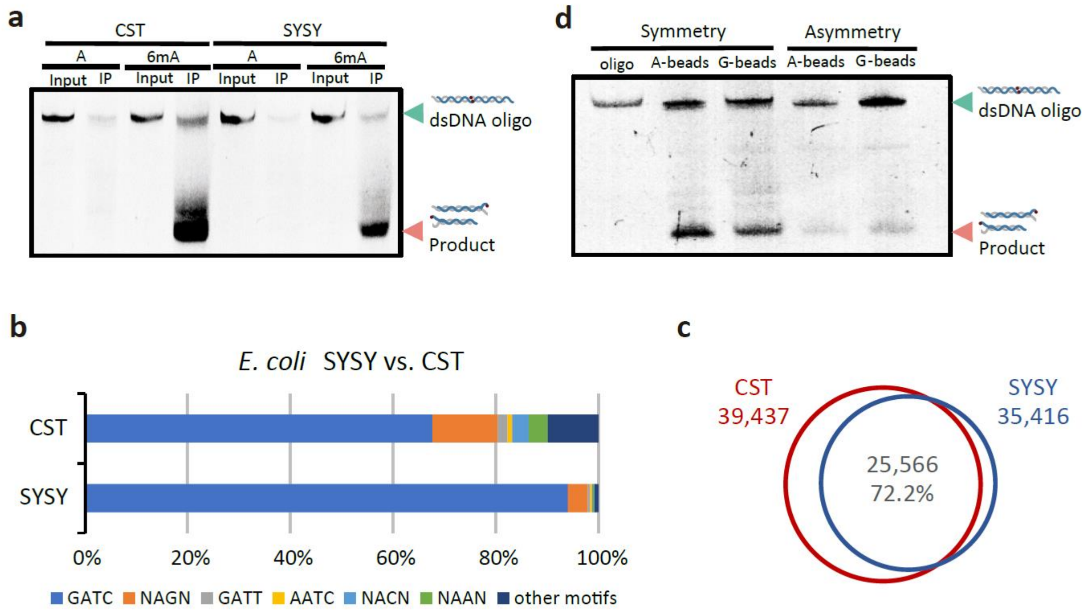

Figure S5. The 6mA distribution and sequencing saturation curves of MM-seq in *E. coli* and *C. reinhardtii*. a) The accumulative plot of distances between 6mA sites of *C. reinhardtii* and transcription start sites (TSS). 6mA sites were detected using lambda exonuclease. 6mA sites within symmetric AT motif and asymmetric 6mA sites are separately plotted. b-e) Saturation curves of *E. coli* and *C. reinhardtii* using T7E1 endonuclease and lambda exonuclease, respectively.

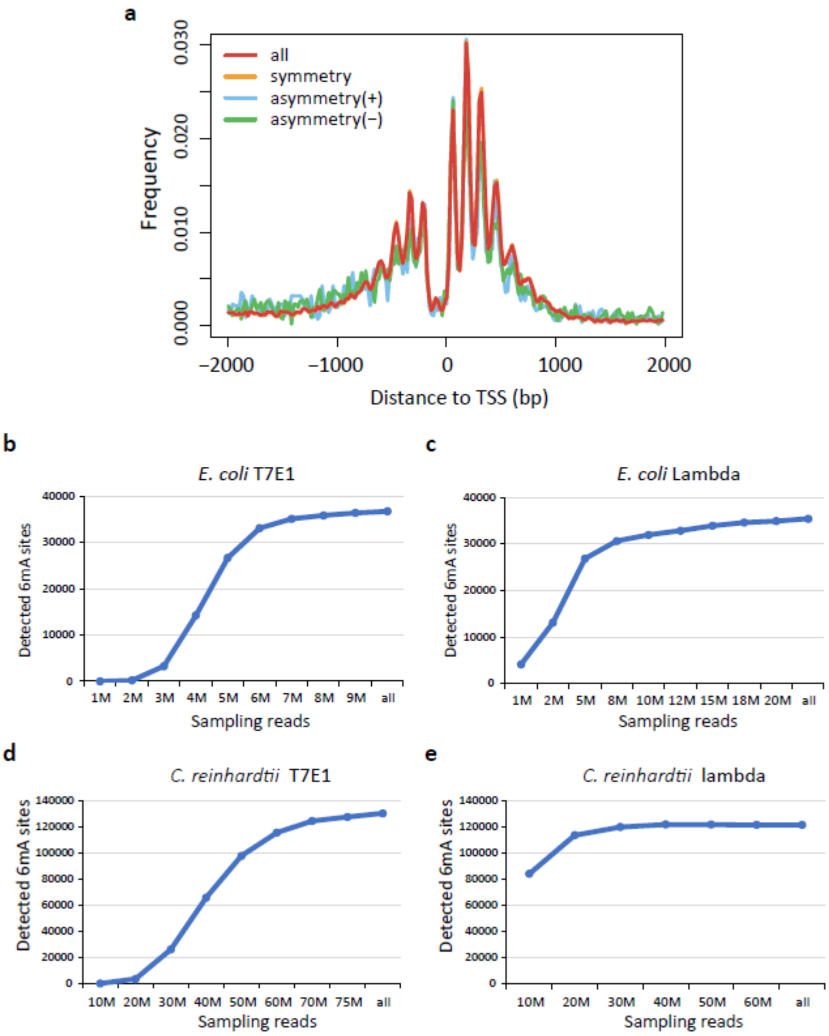

Figure S6. The presence of 5mC sites has little influence for the 6mA detection using MM-seq. a) Intersection of 6mA sites with adjacent 5mC identified in wild-type *E. coli* and that depleted of 5mC. b-c) Digested rates of 5' and 3' terminal for detected 6mA sites.

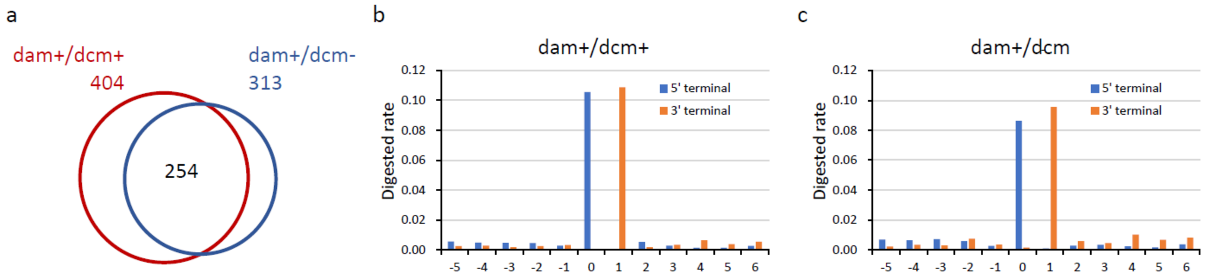

Figure S7. 6mA sites detected by MM-seq in mammalian cells. a-b) Genomic 6mA sites density on each chromosome in HEK293T and mESCs, respectively. c) Accumulative plot for 6mA peaks and single-nucleotide 6mA sites located near TSS.

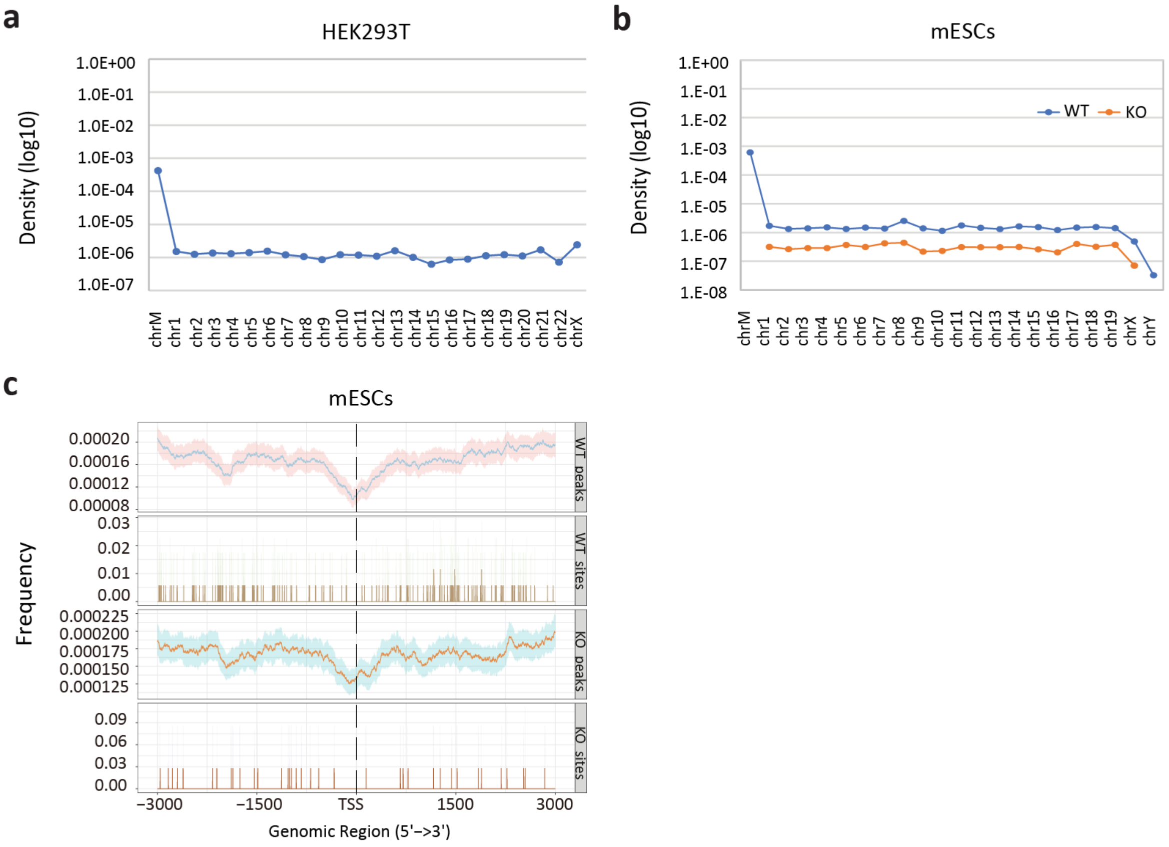

Supplementary Tables

Table S1. Classification of 6mA sites in *Chlamydomonas reinhardtii* identified by MM-seq based on T7E1 and MBN endonucleases.

	**T7E1**		**MBN**	
	**Sites**	**Percentage**	**Sites**	**Percentage**
**Statistically significant sites**	150,133	100.00%	129,899	100.00%
**6mA sites (stop at A/T sites)**	130,457	86.89%	104,791	80.67%
**VATB**	124,833	95.69%	87,070	83.09%
**AT**	125,101	95.89%	88,540	84.49%
**AC**	1,315	1.01%	9,436	9.00%
**AG**	919	0.70%	2,383	2.27%
**AA**	3,122	2.39%	4,432	4.23%

Table S2. Classification of 6mA sites in *E. coli* identified by MM-seq based on T7E1 endonuclease and lambda exonuclease.

	T7E1	Lambda
Statistically significant sites	42,079	50,775
6mA sites (stop at A/T sites)	36,625	35,416
Fully methylated 6mA	-	31,549
Percentage of fully methylated 6mA	-	89.09%
Detected 6mA within GATC	35,035	33,327
Percentage of GATC motif in all detected 6mA	95.66%	94.10%
Sequenced GATC motif >= minimum depths	36,298	36,585
Percentage of GATC motif in sequenced GATC	96.52%	91.09%

Table S3. Classification of 6mA sites in *Chlamydomonas reinhardtii* identified by MM-seq based on Lambda exonuclease.

	Sites	Percentage
**Statistically significant sites**	115,312	100.00%
**6mA sites (stop at A/T sites)**	115,263	99.96%
**AT symmetry**	107,040	92.83%
**6mA asymmetry**	8,223	7.13%
**Asymmetry (+)**	4,074	3.53%
**Asymmetry (-)**	4,149	3.60%
**AT asymmetry**	8,118	7.04%
**AC asymmetry**	23	0.02%
**AG asymmetry**	74	0.06%
**AA asymmetry**	8	0.01%
**6mA (sense)**	57,594	49.95%
**6mA (antisense)**	57,669	50.01%

Table S4. 6mA sites count in METTL4 knockdown 293T cells and control cells by MM-seq using two different antibodies.

	SYSY	CST	shNC	shMettl4
**6mA sites**	4184	3846	3311	4361
**chrM**	5	4	1	1

Table S5. Sequences of synthetic oligos and primers for q-PCR.

ID	Sequence
**DNA A oligo1-FP**	5’-CCTTGTGTCTTCCGGTTCGTGTGTCTTGGATCTCGATTGGTC-3’FAM
**DNA A oligo1-RP**	5’-TAAGCGGAGGCACAAGCGACCAATCGAGATCCAAGACACA-3’FAM
**DNA 6mA oligo1-FP**	5’-CCTTGTGTCTTCCGGTTCGTGTGTCTTGG(6mA)TCTCGATTGGTCGCTTGTGCCTCCGCTTA-3’FAM
**DNA 6mA oligo1-RP**	5’-TAAGCGGAGGCACAAGCGACCAATCGAG(6mA)TCCAAGACACACGAACCGGAAGACACAAGG-3’FAM
**DNA A oligo2-FP**	5’-TTCAGAGCCCAGCTGTGTCAATGCGCATGTGTTGAATGTGGAGACATGTCCCGACAAGTCCACACCCGGACACACAACTT-3’FAM
**DNA A oligo2-RP**	5’-AAGTTGTGTGTCCGGGTGTGGACTTGTCGGGACATGTCTCCACATTCAACACATGCGCATTGACACAGCTGGGCTCTGAA-3’FAM
**DNA 6mA oligo2-FP**	5’-TTCAGAGCCCAGCTGTGTCAATGCGC(6mA)TGTGTTGA(6mA)TGTGGAGAC(6mA)TGTCCCGACAAGTCCACACCCGGACACACAACTT-3’FAM
**DNA 6mA oligo2-RP**	5’-AAGTTGTGTGTCCGGGTGTGGACTTGTCGGGAC(6mA)TGTCTCCAC(6mA)TTCAACAC(6mA)TGCGCATTGACACAGCTGGGCTCTGAA-3’FAM
**GAPDH-FP**	TGAGTACGTCGTGGAGTCCA
**GAPDH-RP**	TTCACACCCATGACGAACAT
**DNA-mismatch1 site-FP**	5’-CCTTGTGTCTTCCGGTTCGTGTGTCTTGGcTCTAGATTGGTC-3’FAM
**DNA-mismatch2 site-FP**	5’-CCTTGTGTCTTCCGGTTCGTGTGTCTTGGcgCTAGATTGGTC-3’FAM
**Sh*METTL4***	CCGGGCAAATACCTATCCCTAAATTCTCGAGAATTTAGGGATAGGTATTTGCTTTTTG

Table S6. Mapping ratio towards mycoplasma genome of sequencing reads from each sample.

**Sample**	**mapping ratio**
293_WT_CST	0.04%
293_WT_SYSY	0.09%
293_shMettl4_SYSY	0.16%
293_shNC_SYSY	0.09%
293_WT	0.00%
293_WGA	0.00%
mESC_WT	0.00%
mESC_M3KO	0.00%
